# Satellite-derived sandy shoreline trends and interannual variability along the Atlantic coast of Europe

**DOI:** 10.1038/s41598-024-63849-4

**Published:** 2024-06-06

**Authors:** Bruno Castelle, Etiënne Kras, Gerd Masselink, Tim Scott, Aikaterini Konstantinou, Arjen Luijendijk

**Affiliations:** 1 Univ. Bordeaux, CNRS, Bordeaux INP, EPOC UMR 5805, 33600 Pessac, France; 2 Deltares, Boussinesqweg, 2629 HV Delft, The Netherlands; 3 Coastal Processes Research Group, School of Biological and Marine Sciences, University of Plymouth, Plymouth, PL4 8AA UK; 4 Delft University of Technology, Stevinweg, 2628 CN Delft, The Netherlands

**Keywords:** Sandy coast, Climate indices, Satellite-derived shoreline, Long-term erosion, Interannual variability, Coastal settings, Natural hazards, Physical oceanography

## Abstract

Monitoring sandy shoreline evolution from years to decades is critical to understand the past and predict the future of our coasts. Optical satellite imagery can now infer such datasets globally, but sometimes with large uncertainties, poor spatial resolution, and thus debatable outcomes. Here we validate and analyse satellite-derived-shoreline positions (1984–2021) along the Atlantic coast of Europe using a moving-averaged approach based on coastline characteristics, indicating conservative uncertainties of long-term trends around 0.4 m/year and a potential bias towards accretion. We show that west-facing open coasts are more prone to long-term erosion, whereas relatively closed coasts favor accretion, although most of computed trends fall within the range of uncertainty. Interannual shoreline variability is influenced by regionally dominant atmospheric climate indices. Quasi-straight open coastlines typically show the strongest and more alongshore-uniform links, while embayed coastlines, especially those not exposed to the dominant wave climate, show weaker and more variable correlation with the indices. Our results provide a spatial continuum between previous local-scale studies, while emphasizing the necessity to further reduce satellite-derived shoreline trend uncertainties. They also call for applications based on a relevant averaging approach and the inclusion of coastal setting parameters to unravel the forcing-response spectrum of sandy shorelines globally.

## Introduction

Sandy shorelines, which cover approximately one third of the Earth’s ice-free coastline^1^, provide important natural^2^ and socio-economical^3^ resources. They are also amongst the world’s most energetic and dynamic environments^4^ and in the long term they are threatened by climate change and declining sediment supply^5^. It is thus critical to improve our understanding and predictive capacity of shoreline evolution over a broad range of timescales spanning days-to-century, to support the development and sustainability of sandy coastal environments^6^. Past multidecadal shoreline trends can be extrapolated to provide insight into future shoreline positions at the 2100 horizon (e.g.^7,8^), while interannual shoreline variability will typically dominate the shoreline signal and its uncertainties during the next few decades before sea level rise takes over (e.g.^9^). Such interannual shoreline variability is often primarily enforced by large-scale climate patterns of atmospheric or coupled ocean-atmospheric variability (e.g. El Niño-Southern Oscillation (ENSO) and North Atlantic Oscillation (NAO)^10,11^). The seasonal to decadal predictability of these climate patterns recently showed increasing skill^12,13^, which may allow a reduction in shoreline evolution uncertainties in the next decades. Understanding shoreline evolution on inter-annual to decadal timescales, and at local to regional spatial scales is therefore critical to understand the past and predict the future of our coasts.

Until recently, observation of coastal change on decadal/multi-decadal timescales at a sufficient temporal resolution (e.g. days, month) was only available at a small number of well-monitored sites using Global Navigation Satellite Systems (GNSS) surveys and/or video monitoring techniques^14–20^. Within only a few years satellite remote sensing has transformed decadal timescale coastal science from a data-poor into a data-rich field (see literature review in^21^). In particular, free-of-charge publicly-available optical satellite imagery can now be used to derive shoreline positions on large spatial ($\mathcal {O}$(1000) m to global) and temporal ($\mathcal {O}$(10) years) scales at relatively high frequency ($\mathcal {O}$(1–10) days) using a wealth of techniques (e.g.^22–25^). Luijendijk et al.^1^ first provided a high spatial (transect spacing ranging approximately 200–500 m) and temporal (yearly) resolution Satellite-Derived Shoreline (SDS) dataset at global scale, focusing on long-term shoreline trends, offering fresh perspectives of increased understanding of shoreline change globally^26^. However, although SDS uncertainties are typically around 10–15 m on many beaches (e.g.^22^), SDS accuracy dramatically worsens on high-energy and/or low-gradient and/or meso-macrotidal beaches^27,28^. Water-level (including wave action) correction^27,28^ can be applied to reduce uncertainties. However, it cannot be applied globally because the type of water-level correction depends on the beach state^28^ and breaking wave conditions, which are not available along the global coastline. Spatial and temporal averaging of uncertain SDS datasets can be performed to filter out some of the SDS noise and to further provide fair insight into the spatial and temporal modes of shoreline variability^29,30^. Although such an approach can work on relatively straight stretches of coast, it is challenging in other environments such as embayed beaches, sandspits or estuary mouths where the time and space patterns of shoreline change can strongly vary alongshore. Because of some of these limitations, there has been a growing number of concerns raised by the coastal science community (e.g.^5,31–33^) on global applications where satellite-derived data, including SDS, are used to provide debatable conclusions on the past or future of our coasts globally. A recent example emphasized by^34^ is the use of coarse shoreline transect spacing of up to 0.5$^\circ$ to address shoreline change globally, which does not sample the great diversity of coastal settings, behaviors, and geomorphic changes. Another limitation of previous SDS studies at regional to global scales is that shoreline change characteristics are typically averaged geographically (i.e. latitude, longitude, country, continent^1,35^), even with very coarse resolution transects^36^. A large body of literature based on field data shows that coastal settings, such as coastline orientation with respect to the dominant wave climate (e.g.^37^) and/or wave sheltering from major headlands or offshore islands^38,39^ is crucial to the spatial and temporal modes of shoreline response.

It is unclear if, and to what extent, a global SDS dataset can be used to provide a robust estimation of long-term trends, to identify the primary climate modes of atmospheric variability affecting interannual shoreline change, and to provide new insights into the spatial variability of these controls depending on some basic coastal settings. Here we focus along the Atlantic coast of Europe because: (1) the large waves^40^ and tides^41^ challenge SDS accuracy^27,28^ and thus provide a conservative assessment for global SDS applications; (2) it comprises a large variability of coastal settings with long sandy barriers, embayed beaches, estuary mouths and tidal inlets, with also a large variability in terms of coastline orientation; (3) it contains some of the most monitored and studied stretches of coast in the world^18–20,37^. The Atlantic coast of Europe is exposed to high-energy ocean waves generated in the North Atlantic Ocean with trends and climate controls which have been identified locally already (e.g.^29,33,37,42–44^), and with such previous work providing critical information to interpret and validate our findings derived from optical satellite imagery.

In this contribution, we validate and consult an improved state-of-the-art global SDS dataset to address the spatial distribution of long-term trends and interannual variability of sandy shores along the Atlantic coast of Europe, and to further identify the primary drivers and coastal settings affecting this spatial variability. By applying a moving-average approach based on distance and coastline orientation, we show that west-facing fully-exposed coasts are more affected by long-term erosion, with interannual shoreline variability controlled by the North Atlantic Oscillation (NAO^10^) at the most northern $\gtrsim 50\,^\circ$N and southern $\lesssim 40\,^\circ$N extents, and in between by the West Europe Pressure Anomaly (WEPA^42^), which is a climate index developed specifically to address wave climate variability along the Atlantic coast of Europe. In contrast, relatively closed sandy coastlines tend to be more prone to long-term accretion on average, and coastlines not fully exposed to the dominant ocean waves show complex and variable correlations with the dominant climate indices, providing a spatial continuum between previous local-scale studies. While recognizing the uncertainties associated with satellite-derived shoreline analysis, we advocate that geographically-averaged SDS analyses, especially based on coarse transect spacing ($\mathcal {O}$(1–10) km), can miss crucial information on the drivers and coastal settings affecting shoreline variability and trend, and that future global SDS analysis will benefit from including such information at high spatial resolution to robustly cluster forcing-response shoreline modes.

## Results

### Study site characteristics

**Figure 1:**
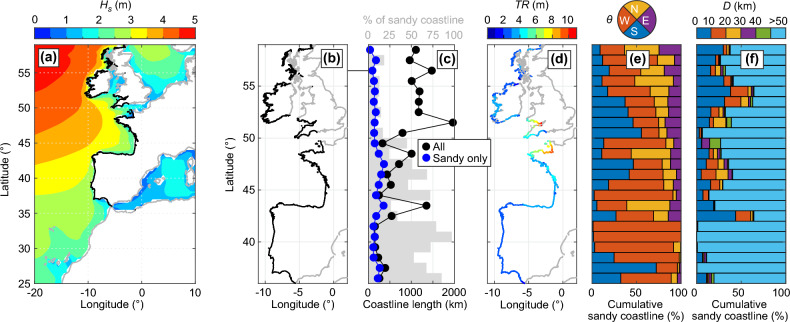
Latitudinal distribution of satellite-derived sandy coastline characteristics. (**a**) Winter (DJFM)-mean significant wave height $H_s$ from ERA5 global reanalysis and coastline of interest (thick black line) which is zoomed onto in (**b**). (**c**) Latitudinal distribution (binned at 1$^\circ$ interval) of the length of total (black dots) and sandy only (blue dots) coastline and its corresponding percentage (grey bars). (**d**,**e**) Sandy coastline characteristics with (**d**) mapped average tidal range *TR* and latitudinal distribution (binned at 1$^\circ$ interval) of (**e**) sandy coastline orientation $\theta$, with W and N (E and S) facing coastlines being relatively exposed to (sheltered from) incident ocean waves, and of (**e**) sandy coastline orthogonal distance *D* to the closest coast, with $D>50$ km and $D<50$ km referring to relatively open and closed sandy coasts, respectively.

The study area covers the west coast of Europe (Fig. 1a,b) which is exposed to high-energy waves generated in the North Atlantic Ocean. We used the global Shoreline Monitor (SM) yearly SDS dataset made of approximately 200- to 500-m spaced transects^1^. This dataset was extended to 2021 (1984–2021 coverage) and used an improved sandy (including gravel) shoreline classification (see “Methods”^45^). In total, the study area covers approximately 11,000 km of coastline, including approximately 2840 km of sandy shores ($\approx 25.8\%$). Such distribution largely varies latitudinally (Fig. 1c) with, overall, a larger proportion of sandy coastlines in the south ($<50\,^\circ$N, $\approx 46.6$% of sandy shores), than in the north (exposed coast of UK at $>50\,^\circ$N, $\approx 10.7$% of sandy shores), highlighting the difference of overall coastal typology between the British Isles and the continental beaches. Average tide range *TR* at the coast computed (from Copernicus Climate Change Service (C3S)^46^), which is critical to SDS uncertainties, also shows large spatial variability ranging from 0.96 m along the southwest Scottish coast, to 10.73 m inside the Bristol Channel, with a mean of 3.49 m (Fig. 1d) highlighting the large tidal amplitude along the study area. Winter-wave climate is highly energetic with, based on C3S ERA5 reanalysis^47^, winter wave height increasing northwards and winter-mean significant wave height exceeding 4 m offshore the Irish and Scottish coasts (Fig. 1a). Hereafter, only the sandy coast part of the study area is analysed. West-facing sandy coasts are dominant ($\approx$ 1380 km, 48.5%), followed by south-facing ($\approx$ 760 km, 26.7%), north-facing ($\approx$ 460 km, 16.1%) and east-facing ($\approx$ 250 km, 8.7%). West-facing open coastlines are particularly represented in southwest France and western coast of Portugal (Fig. 1d). A substantial proportion of sandy shores ($\approx$ 750 km, 26.5%) are relatively closed ($D<$ 50 km, where *D* is the orthogonal distance to the closest coast, see “Methods”), and are primarily located in northwest Spain, west-northwest France and UK (Fig. 1e). Important to the SDS analysis is the amount of satellite images used to generate the yearly composite and further compute shoreline position. The number of individual images used in a yearly composite image and the number of locally missing yearly composite decrease and increase northwards, respectively, due mostly to increased cloud cover^48^, indicating that the accuracy of SDS time series decreases at the highest latitudes.

### Long-term shoreline trends

**Figure 2:**
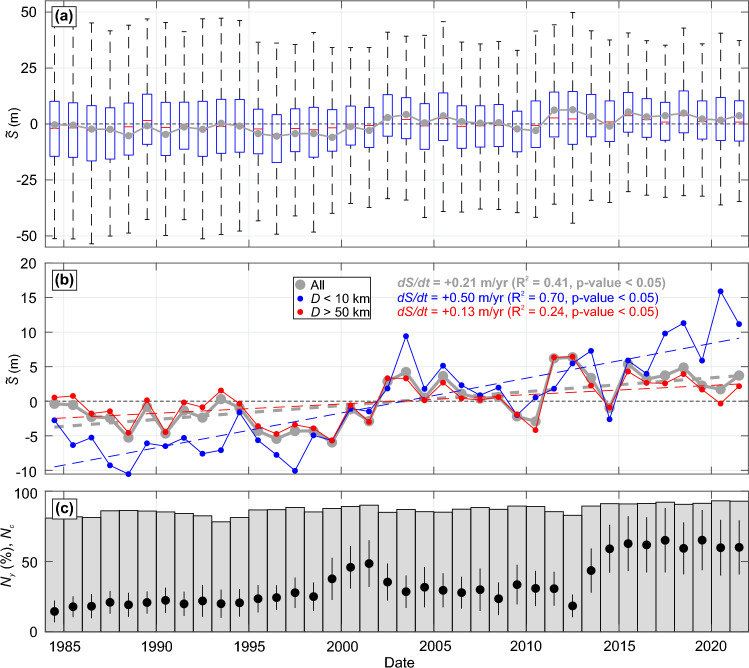
Long-term sandy shoreline trend and influence of coastline sheltering. (**a**) Time series of spatially-averaged shoreline position around the mean $\widetilde{S}$ for the entire sandy coast (thick grey) and corresponding box plot with the central horizontal red mark indicating the median, the bottom and top edges of the box indicating the 25th and 75th percentiles, respectively, and the whisker length indicating 1.5 times the interquantile range, which shows relatively well normally distribute $\widetilde{S}$. (**b**) Same time series of spatially-averaged shoreline position around the mean $\widetilde{S}$ for the entire sandy coast (thick grey), and focusing on significantly closed ($D<10$ km, blue) and relatively open ($D>50$ km, red) coasts. The corresponding long-term trends *dS*/*dt* are also shown (dashed lines) and quantified using the same color scheme. (**c**) Corresponding time series of SM SDS spatial coverage $N_y$ ($\%$, bars) and number of images used per yearly composite $N_c$ with the dots and vertical bars representing the mean and ± standard deviation, respectively.

Figure 2a,b shows, averaged over study area sandy coastline, the time series of yearly shoreline deviation around the long-term (1984–2021) mean $\tilde{S}$, together with the evolution of the yearly-mean SM SDS spatial coverage $N_y$ and of the number of images used per yearly composite $N_c$ (Fig. 2c). Accounting for all the sandy shorelines, and despite interannual shoreline variability of $\mathcal {O}$(1 m), a statistically-significant (p-value < 0.05) accreting trend of + 0.21(±0.4) m/year is found (see section “SDS post-processing” for the long-term trend uncertainty estimation). This accreting trend is more than doubled if only significantly closed shorelines ($D<10$ km) are considered (+ 0.50(±0.4) m/year, blue line in Fig. 2a), and is almost halved, but still statistically significant (p-value < 0.05), considering relatively open coasts (+ 0.13(±0.4) m/year for $D>50$ km, red line in Fig. 2a). Note that despite different long-term trends, the relatively open and closed shorelines show coherent response (correlation *R* = 0.65 between the blue and red lines in Fig. 2b). Given the long-term trend uncertainties and potential bias towards accretion (see section “Shoreline Monitor (SM) dataset”), these unexpected and controversial results that satellite-derived shorelines along sandy coasts may tend to accrete on average are discussed and tempered later in the Discussion Section.

**Figure 3:**
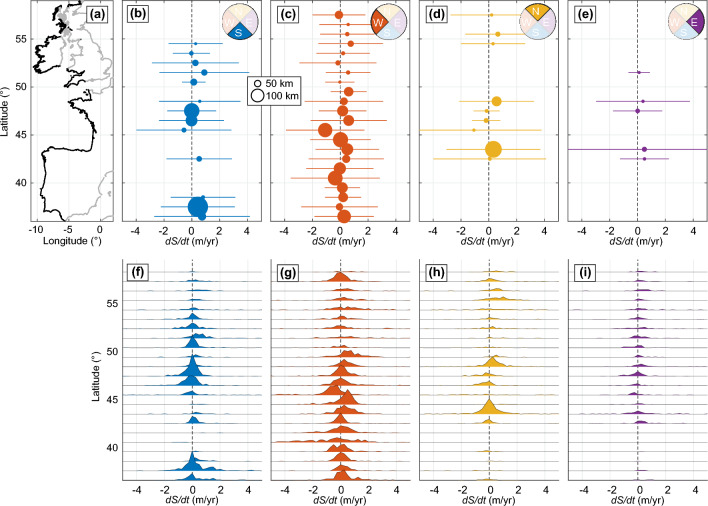
Influence of coastline orientation on sandy shoreline long-term trends. (**a**) Study area coastline (black line) and latitudinal distribution of long-term shoreline trends *dS*/*dt* discriminated by coastline orientation $\theta$, (**b**) south-facing, (**c**) west-facing, (**d**) north-facing and (**e**) east-facing. In (**b**–**e**) only sectors with a cumulative shoreline length larger than 20 km were plotted, the horizontal bars indicate the ± standard deviation of individual transect (no space averaging) long-term trend, and the bubble size is proportional to the cumulative sandy shoreline length. (**f**–**i**) Corresponding latitudinally-binned distribution of long-term shoreline trends.

On average, east-facing (*dS*/*dt* = + 0.33(±0.4) m/year), south-facing (*dS*/*dt* = + 0.29(±0.4) m/year) and north-facing (*dS*/*dt* = + 0.26(±0.4) m/year) coastal stretches are more prone to long-term accretion, although still within the range of uncertainty. A slight overall accretive trend is found (*dS*/*dt* = + 0.13(±0.4) m/year) for west-facing coasts, i.e. an average rate smaller than for all the other coastline orientations. Figure 3 further shows that there is a large latitudinal variability of shoreline trends. The weaker long-term accreting trend of the west-facing coastlines is largely due to the eroding, fully exposed, sandy coasts of southwest France around the Gironde estuary and Maumusson inlet at 45$^\circ<$ latitude $<46^\circ$, the west coast of Portugal and, to a lesser extent, the southwest coast of the UK (Fig. 3b–e). Importantly, the mean long-term trends are very small compared to the regional variability as indicated by the large standard deviation of shoreline trends (horizontal bars in Fig. 3b–e). Despite this large variability, the shoreline change rates are relatively well normally distributed for all coastline orientations and latitudes (Fig. 3f–i).

### Interannual shoreline variability and climates modes of atmospheric variability

**Figure 4:**
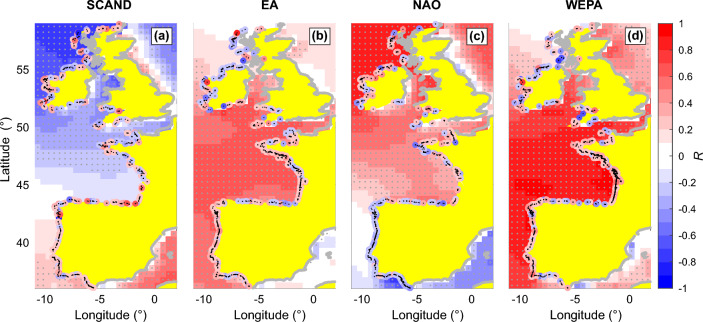
Correlation of winter-mean significant wave height and shoreline change against dominant winter climate indices. Spatial correlation of the winter-mean $H_s$ and yearly shoreline change *dS* against (December to March) winter-averaged climate indices over 1984–2021: (**a**) SCAND; (**b**) EA; (**c**) NAO and (**d**) WEPA. In all panels, for clarity only the shoreline points with a statistically-significant correlation at a 80% confidence level have been plotted, while the grey dots on the wave field show statistically-significant correlations at the 95% confidence level.

Figure 4 shows the spatial correlation *R* of the winter-mean significant wave height $H_s$ and yearly shoreline change *dS* against the primary (December to March) winter-averaged climate indices in the region over the entire time series 1984–2021. Noteworthy, because yearly composites include subsequent spring, summer and early autumn recovery, correlations are expected to be much lower than with post-winter shoreline position as demonstrated by^33^ using in situ shoreline time series. The Scandinavian pattern (SCAND) shows relatively poor correlation with winter wave activity, except at the most northern latitudes along the northern Ireland and Scottish coasts where *R* can exceed 0.7 (Fig. 4a). In contrast, the NAO shows larger positive and negative correlation with winter-mean wave height at the most northern and southern latitudes, respectively, with weak correlation in the transition area. In this transition area (approximately between 38$^\circ$N and 51$^\circ$N), WEPA (Fig. 4d), and to a lesser extent the East Atlantic pattern (EA, Fig. 4b) show a strong positive correlation. The same analysis was performed for yearly shoreline change *dS*. In order to reduce shoreline change uncertainties and be able to robustly address complex coastline shapes without introducing errors, *dS* was averaged using a 5-km moving-average window (*L*) and a coastline orientation $\delta \theta$ = 45$^\circ$ relative to each transect (see “Methods”). Shoreline response shows weaker correlation with primary winter-averaged climate indices and more complex patterns than for winter wave conditions. For instance, a consistent positive correlation with WEPA (meaning that a positive WEPA results in increased erosion) is found along the open sandy coast of southwest France, whereas along more complex shorelines positive and negative correlations typically alternate in space and local scale patterns are mostly difficult to pick up.

**Figure 5:**
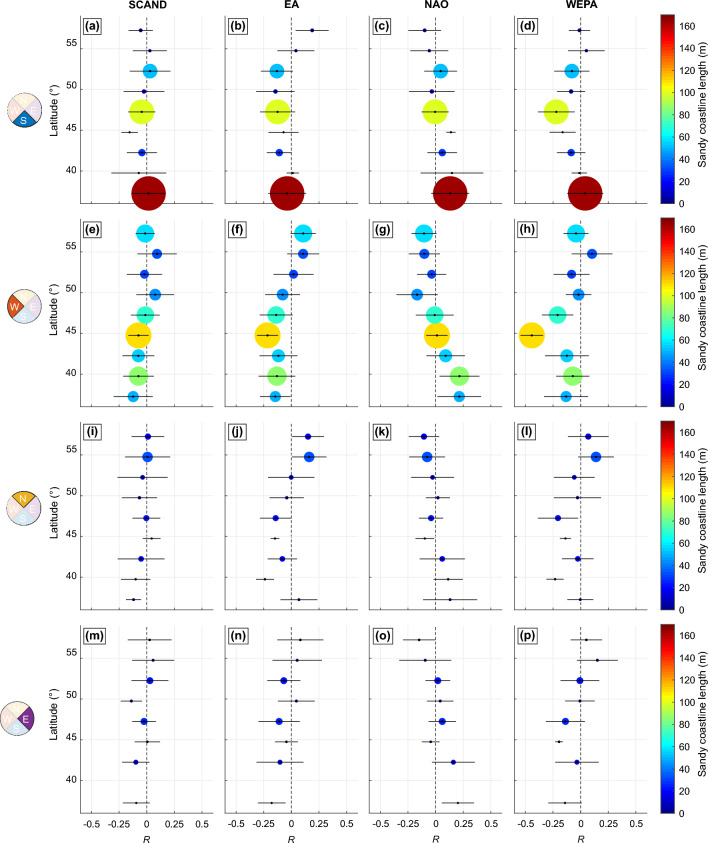
Latitudinal distribution of correlation of shoreline change against dominant winter climate indices and influence of coastline orientation. Latitudinal distribution (2.5$^\circ$ bins) of yearly shoreline change *dS* correlation against the primary winter-mean climate indices (columns) for different coastline orientations (rows) over 1984–2021. In all panels, the bubble size and color indicate the cumulative sandy coastline length, and the horizontal bars indicate the ± standard deviation.

To provide a broader insight into the correlations between the climate indices and shoreline response, Fig. 5 shows the average correlation binned at 2.5$^\circ$ intervals for different coastline orientations. While SCAND does not show any correlation pattern, a clear latitudinal gradient is found with the NAO for the west-facing coasts (Fig. 5g), which is more subtle for the other coastline orientations. Also in line with the wave climate, WEPA, and to a lesser extent EA, shows positive correlation in the NAO-transition zone (Fig. 5f,h). Similar WEPA patterns are found for the south-facing coastlines (Fig. 5d), which are more subtle for the east- and north-facing coastlines. Importantly, except for the west-facing southwest coast of France, bin-averaged correlations are weak (< 0.5), which will be discussed in the next section.

## Discussion

Our results suggest that, on average, sandy SDS may have been accreting (+ 0.21(±0.4) m/year) over the last nearly 40 years along the exposed Atlantic coast of Europe (Fig. 2). This finding goes against the many local observations showing eroding sandy shorelines along the Atlantic coast of Europe. However, many of these works investigated the Portuguese sandy coast (e.g.^49,50^) or the southwest Coast of France^51,52^, which were also found to mostly erode in our dataset (Fig. 4). There is also evidence that many embayed beaches in e.g. France and the UK, are dominated by shoreline rotation and/or show no significant long-term eroding trend^19,20^. Some accreting sectors found here have also been identified in the field (e.g.^53^). The SDS dataset used herein approximately corresponds to the Mean Sea Level (MSL) shoreline proxy which behaviour can contrast with the dune foot shoreline proxy, a relevant shoreline proxy along sandy coasts, due sediment exchanges and redistribution between the dune and the intertidal beach. This is particularly true on meso-macrotidal beaches, which are ubiquitous along the Atlantic coast of Europe (Fig. 1d). This was evidenced by^54^ on a beach in French Britany showing MSL and dune foot shorelines showing opposite behaviour, i.e. eroding dune versus accreting MSL shoreline. Moreover, the SM SDS long-term trend uncertainties were estimated using high-frequency SDS from other approaches^22^ and field data (see Figs. 8 and 10 and section “Methods”), as well as trends computed along rocky shorelines (see section “Methods”). It shows that the computed trends mostly fall within the uncertainty range, with a potential bias towards accretion. Therefore, more work is required to provide more detailed and validated satellite-derived insights into the spatial distribution of long-term eroding and accreting sandy coasts. Such work should involve more validation with field data to better estimate long-term trend uncertainties at a wide range of environments. In addition, progressively improving image composite quality^55^ and modification of certain optical index thresholds between the different satellite missions (e.g.^56^) may explain the long-term trend bias towards accretion. Such bias was identified with Sentinel images (see “Methods”), which were thus removed from the yearly composites used herein. Such impact of evolving satellite missions needs to be explored further for SDS data, especially as such long-term trends can be extrapolated to predict the future of our coast (e.g.^7^).

While previous work essentially geographically averaged shoreline response^1,35,36^, here we investigated the influence of some coastline characteristics, namely the orthogonal distance to the closest coastline *D* and coastline orientation $\theta$, on shoreline trend and response. This allowed us to demonstrate that west-facing, i.e. fully exposed to the dominant incidence of ocean waves, and relatively open ($D>50$ km) sandy coasts are more prone to long-term coastal erosion, with stronger relations between interannual shoreline change and winter climate indices. This is in agreement with local studies showing that enclosed/embayed beaches are less prone to erosion than open beaches^53^. Noteworthy, generalising such finding globally is misleading, as some long open coast sectors are known to accrete at a substantially large rate (e.g. Northern California^30^). Instead, we advocate that such averaging approach through e.g. coastline orientation and/or orthogonal distance to the closest coastline^57^, but also potentially other shoreline characteristics, should help to better understand shoreline response at regional to global scale. We also anticipate that the absence of relatively strong long-term erosion trend of the Atlantic sandy coast of Europe is because west-facing open ($D>50$ km) sandy coasts, which are more prone to erosion, occupy less than half (39.5%, with a long-term trend of + 0.03(±0.4) m/year) of the total coastline and because of the absence of deltaic coastlines, many of which erode globally^58,59^, and which may contribute disproportionally to global averages. Finally, it must be acknowledged that the extension and revision of the SM SDS dataset used herein (see “Methods”) largely reduced the proportion of classified sandy shores^1,7^, and also reduced the average accretion trends along the Atlantic coast of Europe compared to^1^. Future global shoreline long-term trend analyses and extrapolation in the future will need to be updated with such improved shoreline classification and using more robust past long-term trend computations.

Contrary to some previous work (e.g.^36^), we addressed the correlation between different climate indices against the yearly *change* in shoreline position *dS*^29,60^, not against the yearly-mean shoreline position *S*. Indeed, addressing correlation between shoreline position *S* and a climate index assumes a linear response of the shoreline position to incident wave conditions, which is against fundamental understanding of beach and shoreline response^4,61^, and against field evidence on many coasts^33,62^. Another relevant approach could have been to compute the anomalies in shoreline position during the prolonged positive and negative phases of the different climate indices, similar to^35^ for prolonged positive and negative ENSO phase for the Pacific Ocean coast. However, given the complex interplay between the different dominant climate indices for the Atlantic coast and their lack of multi-annual periodicity and persistence, a systematic comparison between the yearly shoreline change *dS* and the winter climate indices was preferred. Finally, only winter (December to March) climate indices were used here, which is based on field evidence that winter wave conditions control interannual shoreline variability at many sandy coast environments along the Atlantic coast of Europe^43^.

The latitudinal distribution of correlations between shoreline and the climate indices (Fig. 5) are in line with the spatial correlation maps of the winter-mean significant wave height (Fig. 4). Correlation maps with winter-mean significant wave height are essentially in line with previous work^42,62^. However, the details around some sheltered and protected areas, which are typically characterised by multi-directional wave climates, are not reproduced as they require high-resolution wave modelling^63^. We found that, particularly along west-facing coastlines, shoreline response is positively (negatively) correlated against NAO at the highest (lowest) latitudes, meaning that positive (negative) NAO results in increased (decreased) winter erosion. This is in line with local observation in Northern Ireland^40^ and south Spain^64^. In between, WEPA and to a lesser extent EA, is positively correlated with shoreline response, which is also in line with a wealth of observations (e.g.^29,37,43,44,51,65,66^). The impact of the outstanding winter of 2013/2014^40^ is also relatively well captured in the time series of the mean shoreline position (Fig. 2), with sediment redistribution between the dune and the intertidal beach^54^ assumed to smooth the signature of this winter on the MSL shoreline.

**Figure 6:**
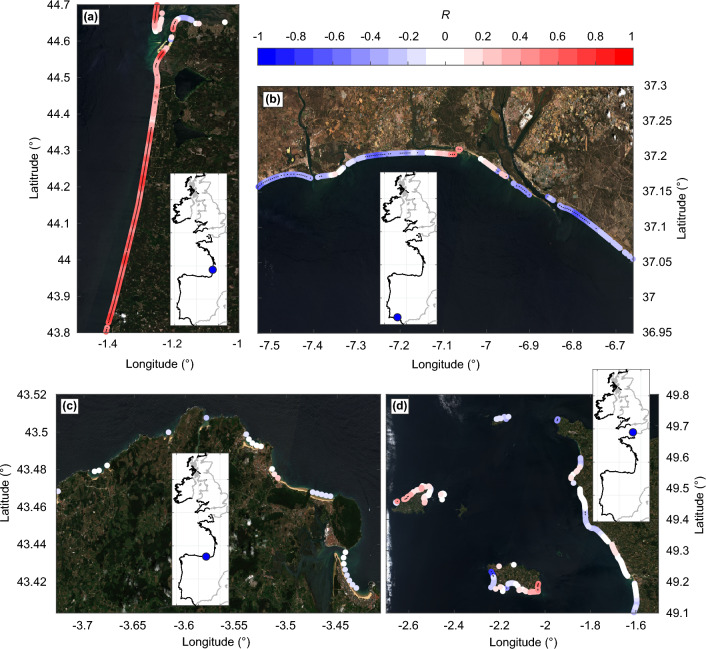
Influence of coastal settings on correlation of shoreline change against winter climate indices. Zoom onto spatial correlation of yearly shoreline change *dS* against climate indices on open coast sectors over 2008–2021: (**a**) WEPA, Landes coast, southwest France and (**b**) NAO, southwest Spain and embayed coast sectors over 2000–2021: (**c**) WEPA, Sector of the Cantabria coast, north Spain and (**d**) WEPA, Cotentin peninsula, northwest France.

Latitudinally-binned average correlations are mostly weak (Fig. 5), but show clear latitudinal distribution for WEPA and NAO, especially for the west-facing beaches. Correlations were also computed for different time periods (not shown here), showing similar patterns and thus providing confidence in the overall patterns. Only quasi-straight open coast beaches show relatively alongshore-uniform statistically significant correlation. This is illustrated in Fig. 6a for the period 2008–2021 for the southwest coast of France, with a positive correlation against WEPA except close to the tidal inlet of Arcachon, and in Fig. 6b for the south coast of Spain with a negative correlation against NAO, except once again close to inlets and structures. Such weak correlation is also observed with field data, primarily because of the influence of the antecedent morphology (memory effects) on winter erosion^33^. Another reason why correlations are weak is that, in line with previous work (e.g.^27,36^), correlations were computed here for each individual climate index, i.e. without exploring the interactions between these large-scale climate patterns. However, interactions of these teleconnections can play an important role in storminess, and thus winter wave conditions and shoreline response, as for instance evidenced with NAO, EA and SCAN for wind speed with Europe^67^. More advance analysis accounting for the interaction of overlapping phases of the different large-scale climate patterns should be considered in future work. In contrast with the relatively alongshore-uniform statistically significant correlation found along quasi-straight open coast beaches, along a substantial amount of small coastal embayments, correlations are weak (Fig. 6c,d) and sometimes not fully in line with previous work based on high-resolution data^66^. Along such embayed coastlines, shoreline response can result from the complex response of multiple atmospheric indices^33,44^, which can explain the weaker correlation. Future work should explore the links between interannual shoreline response and different combinations of multiple atmospheric indices. In addition, anthropogenic impacts, such as implementation of coastal structures, beach nourishments and coastal land reclamation can locally largely impact interannual shoreline response and long-term trends, but were disregarded here. Given that image composites were used here, water level corrections to reduce shoreline uncertainties were not applied. However, as shown by^35^ waterlines have temporal biases at a range of scales for transects across the globe. Image yearly composites made of tens of images (see e.g. Fig. 2c) can reduce such biases. Further, as shown along the southwest coast of France and Pacific coast of the US spatial averaging can reduce shoreline uncertainties and provide reliable shoreline long-term trends and interannual variability even along high-energy tidal coasts^29,30^. We think that such time- and space-averaging, pending a large number of individual images in the composites and reasonably dense transects (spacing of $\mathcal {O}$(100 m)), can be an interesting, yet less accurate, alternative to the tidal-level correction of individual images^25,35^.

The present SDS analysis focused on some of the most energetic environments and with large tide range, thus challenging the SDS accuracy. However, based on a detailed analysis including exposure to the dominant ocean wave direction and discrimination of relatively open and closed coastlines, this work provides new insight into shoreline change from local to regional scale. It also provides a valuable spatial continuum between previous local-scale studies aiming at linking coastal response with large-scale climate patterns of atmospheric variability.

## Methods

### Shoreline Monitor (SM) dataset

**Figure 7:**
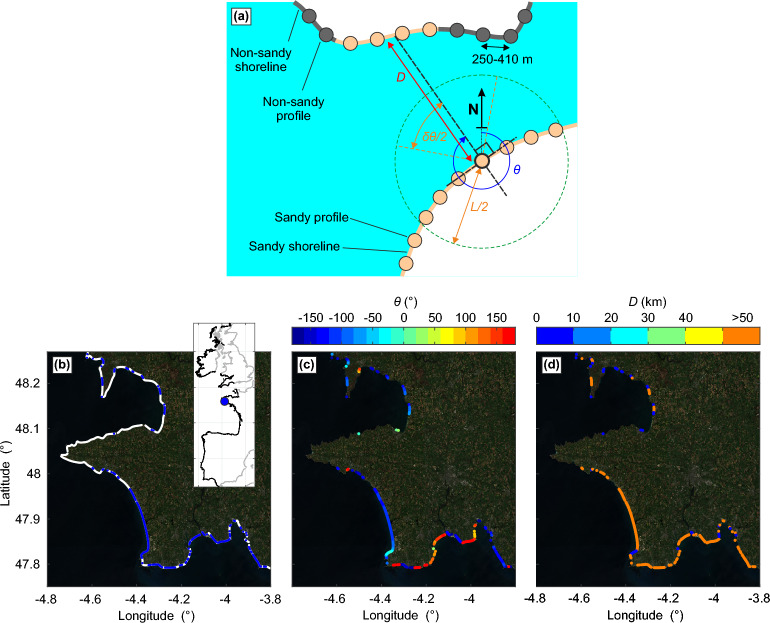
Shoreline Monitor dataset schematics. (**a**) Representation of SM SDS dataset and some variables further used in the analysis: coastline orientation $\theta$, orthogonal distance to the closest coastline *D*, and moving averaged distance *L* considering neighbouring transects with a coastline orientation $\theta$ within $\pm \delta \theta$ with (**b**–**d**) example zoomed onto French Brittany with (**b**) shoreline type (blue: sandy, white: other) and sandy (**c**) coastline orientation $\theta$ and (**d**) orthogonal distance to the closest coastline *D*.

The Shoreline Monitor (SM; http://shorelinemonitor.deltares.nl/) SDS dataset (1984–2016 in^1^) is based on Landsat (5 to 8) and Sentinel-2 (only 2016) yearly composite images and comprises over 2.2 million transects distributed globally (see Fig. 7 for a schematic visualization). It is derived by leveraging the petabyte image catalogue and parallel computing facilities of the Google Earth Engine (GEE)^68^. A thresholding method^69^ was used on yearly Top-Of-Atmosphere (TOA) reflectance composites to remove the effects of noise (clouds and shadows). For each composite image the Normalised Difference Water Index (NDWI) was computed which, combined with the Otsu thresholding method^70^ and a region growing algorithm^71^, provided the most probable threshold to classify water and land on the image. The water line was then smoothed using a 1D Gaussian smoothing operation to obtain shoreline vectors at sub-pixel resolution without the need of supplementing field data^72^. The resulting shoreline approximately matches the MSL contour as the composite image analysis decreases the influence of the tidal stage on the detected shoreline positions.

An updated version of the SM SDS dataset is used in this study. The updated version only contains Landsat (5 to 8) images as a preliminary investigations indicated that inclusion of Sentinel images resulted in a landward shift of shoreline position by approximately 10 m averaged across the entire Atlantic coast of Europe from 2015 onwards. Sentinel images were thus removed from the yearly composites. Furthermore, the dataset is extended up to December 2021 and hence adds another five years of data. The total length of the dataset now equals 38 years. Previously, the years 1990–2000 contained a very low number of usable composite images. Recent updates to the image catalogs of Landsat (and therefore also the updated SDS dataset) increased the available composite images in this period significantly. Besides, the cloud cover threshold is adjusted. This increased the number of available composite images even more. Finally, a new classification of sandy, muddy, and cliff coasts^45^ is added to allow for a better distinction between sandy and other environments. This decreased by approximately 38% the amount of muddy and rocky coastline previously classified as sandy in our study area. Noteworthy, such correction was critical in the UK where 58% of the coasts previously classified as sandy are now correctly identified as rocky using the new classification^45^.

In the present work, only the SM SDS dataset along the western part of Europe was used, from Gibraltar in the South to the northern tip of the Scottish coast. In order to focus on regions which are primarily affected by ocean waves generated in the North Atlantic Ocean^63^, we also disregarded the Irish Sea coastline, the French and UK coastline east of the Cotentin peninsula in the English Channel, and some sheltered and/or east-facing Scottish coastline (see Fig. 1b). This resulted in a total of 34,874 transects, comprising 8281 sandy transects ($\approx 24\%$), which were analysed in the present work. Transects spacing varies as the latitude cosine, ranging for the sandy sectors from 254.8 m in the north to 407.1 m in the south with a mean of 315.5 m. The SM SDS dataset was further processed to compute some other shoreline characteristics (Fig. 7). First, shoreline orientation $\theta$ was computed using the start and end points of each transect. Secondly, for each transect we computed the orthogonal distance *D* to the closest coast. Overall, W and N (E and S) facing coastlines are considered relatively exposed to (sheltered from) incident ocean waves, while $D>50$ km and $D<50$ km typically refer to relatively open and closed sandy coasts, respectively. Because spatial averaging can help to smooth out uncertain, noisy, SDS datasets^29,30^, we also defined a moving average distance *L* considering neighbouring transects with a coastline orientation $\theta$ within $\pm \delta \theta$ (Fig. 7).

### Validation

Luijendijk et al.^1^ already provided a validation of the SM SDS dataset at multiple sites in the world where ground-truth field data are available. However, validation was restricted to sandy coasts with large shoreline variability (amplitude of $\mathcal {O}$(100 m)) and/or a small tidal range. In addition, validation was only performed on long-term trends and interannual variability, which is typically $\mathcal {O}$(1–10 m) on most of sandy coasts, was not addressed. Below, validation is performed along the southwest coast of France, which is a high-energy meso-macrotidal (average tide range of approximately 3 m) environment. This coast is characterised by alongshore-variable shoreline trends^29^ and interannual shoreline variability of $\mathcal {O}$(1–10 m), which is in most locations enforced by climate modes of atmospheric variability (e.g.^29,33,43^). Below, a state-of-the-art SDS dataset is used to validate SM SDS trends along different sectors, before interannual variability is locally validated against field data at a single site (Truc Vert).

#### Long-term shoreline trends and uncertainties

**Figure 8:**
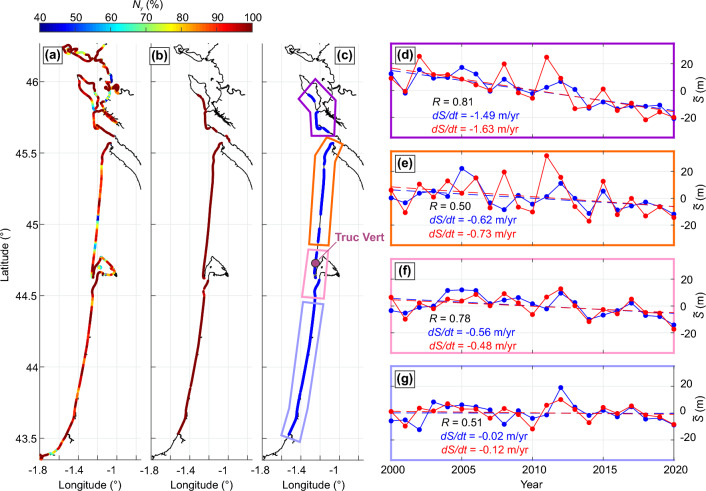
Validation of long-term shoreline trends. Validation of SM SDS trends against CoastSat SDS trends in southwest France. Spatial distribution of the percentage *N* of available yearly composites over 2000–2020 for (**a**) SM and (**b**) CoastSat, with (**c**) the thick blued line indicating transects where CoastSat and SM are both available with $N_y>80\%$ and the coloured polygons showing the areas where the two datasets are compared. (**d**–**g**) Comparison of CoastSat (blue) and SM (red) yearly SDS position deviation around the mean and long-term trends (dashed lines). In each panel, the shoreline trend *dS*/*dt* and correlation coefficient *R* between the two datasets are provided. Truc Vert beach location is indicated in (**c**).

The CoastSat^22,73^ SDS dataset used here for validation is described in^29^ and was averaged yearly for comparison with the SM SDS yearly composites. The period selected was 2000–2020, because prior to 2000 there was a lot of missing years in the CoastSat dataset generated in^29^ before recent updates to the image catalogs of Landsat. Figure 8 shows the validation area and the spatial distribution of the percentage ($N_y$) of available yearly SM (Fig. 8a) and CoastSat (Fig. 8b) SDS data for 2000–2020. In order to perform a fair comparison only the transects with at least 80% ($N_y$) of SDS data availability over 2000–2020 were used (blue shoreline in Fig. 8c). The shoreline time series were further averaged across four different regions (coloured boxes in Fig. 8c) and compared (right-hand panels of Fig. 8). Results show that, using spatially-averaged shoreline transects, the SDS trends of the two datasets are in good agreement with differences around 0.1 m/year for both eroding (Fig. 8d–f) and relatively stable (Fig. 8g) zones. In addition, although performed on a shorter time series, the long-term trends computed here were also compared to shoreline trends computed from in situ measurements at three sites in the UK (Perranporth and two embayment extremities) (2007–2023) and at Truc Vert beach (2003–2023) documented in^33^ and with contrasting long-term trends. Using *L* = 5 km, the SDS (measured) long-term trends are − 0.25 m/year (− 0.14 m/year) at Truc Vert; − 0.68 m/year (− 0.29 m/year) at Perranporth; − 1.27 m/year (− 0.79 m/year) at the southern end of Slapton Sands; + 1.96 m/year (+ 1.03 m/year) at the northern end of Slapton Sands. The weaker long-term trend magnitude at Slapton Sands is explained by the moving averaging (*L* = 5 km), while measurements were collected at the extremities of the embayment where larger changes are observed.

Another approach to estimate the long-term trend uncertainties is to compute the trends on rocky shores only. Across the 5608 km (18,235 transects) of rocky shoreline found along our study area, a mean (median) trend of + 0.27 (+ 0.13) m/year is found with a standard deviation of 1.77 m/year. Although the shoreline detection used in SM is not optimal for rocky coastlines, this result together with the long-term trend comparisons with field data and other satellite products above, indicates conservative uncertainties of long-term trends of sandy shores around 0.4 m/year and a potential bias towards accretion. Of note, this 0.4 m/year uncertainty is also similar to subpixel precision (15 m, over 38 years).

#### Interannual shoreline variability

**Figure 9:**
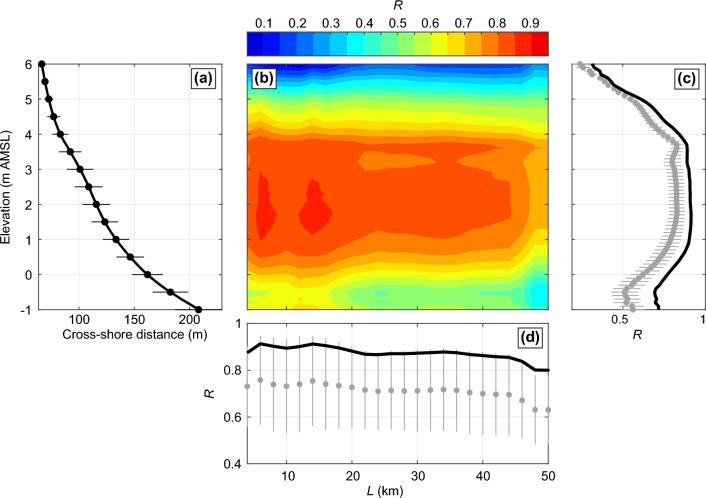
Correlation of interannual shoreline variability against field data for different shoreline proxies and alonghore-averaging windows. Validation of SM SDS interannual variability against in situ surveys over 2003–2022 at Truc Vert beach, southwest France. (**a**) Mean beach profile, with the horizontal lines indicating the ± standard deviation of shoreline position at 0.5-m elevation intervals; (**b**) correlation coefficient *R* (coloured) between SM SDS and yearly-averaged in situ shoreline at Truc Vert, for different in situ shoreline proxy (− 1 m $<z_{prox}<$ 6 m) and different SM alongshore-averaging distance *L*. (**c**,**d**) Corresponding $z_{prox}$-averaged and *L*-averaged correlation *R*, respectively. In (**c**,**d**) the tick black line shows the maximum correlation, and the grey dots show the mean with the grey lines the ± standard deviations.

**Figure 10:**
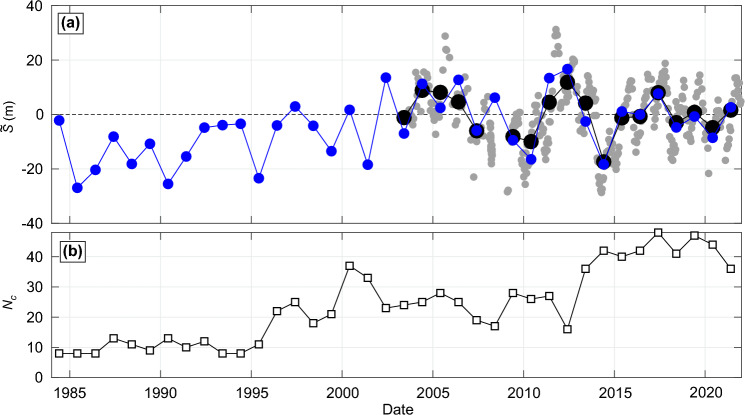
Validation of long-term shoreline change trends. (**a**) Time series of SM SDS (blue line/dots) with a moving-averaged window *L* = 6 km and yearly-averaged shoreline position measured at Truc Vert (black line/dots) from the bimonthly surveys (grey dots) and using shoreline proxy $z_{prox}$ = 1.7 m. (**b**) Time series of the corresponding number of images used for the SM composites.

SM SDS interannual variability was validated against field data collected at Truc Vert beach, France (see location in Fig. 8c), a high-energy west-facing meso-macrotidal (mean *TR* of approximately 3 m) open sandy beach. Since 2003, the beach has been surveyed every 2 or 4 weeks (for a detailed description see^18^). Following earlier work^74,75^, for each survey the shoreline position was defined as the intersection of the alongshore-averaged beach profile with a given elevation proxy $z_{prox}$ above mean sea level (amsl). The alongshore coverage of the surveys progressively increases over time, from approximately 300 m in 2003 to over 2000 m after 2016. We systematically computed the correlation between the SM SDS dataset and measured, yearly-mean, shoreline dataset for different shoreline proxies $z_{prox}$ (at 0.1-m elevation interval) and different alongshore-averaging length *L* (at 1000-m intervals) of the SM SDS data, over the period 2003–2020. Of note, 2008 was removed from the analysis as measurements were only performed in January–February–March before the GNSS system broke down, resulting in an over-eroded measured shoreline in 2008. The resulting correlation matrix is shown in Fig. 9b, with corresponding $z_{prox}$-averaged and *L*-averaged correlations given in Fig. 9c,d, respectively. Correlations are strong and statistically significant on most of the $z_{prox}-L$ spectra, except for the highest part of the profile $z_{prox} \gtrsim 4$ m where the morphology barely moves (Fig. 9a) except during the outstanding winter of 2013/2014. Correlation peaks at 0.91 for *L* = 6 km and $z_{prox}$ = 1.7 m, i.e. slightly above mean high tide level, with the corresponding time series shown in Fig. 10a. For this set of parameters, *R* = 0.91 means that the SM SDS explains over 83% of the observed interannual shoreline variability at Truc Vert. Correlation further increases to 0.93 (87% of the observed shoreline variability) when selecting the more recent period 2013–2022 when the number of images used per yearly composite almost doubled (Fig. 10b).

This validation exercise indicates that the SM SDS dataset can be used to address interannual shoreline variability pending a well-adapted moving average window *L*. The correlation is not maximised for $z_{prox}$ = 0, which is approximately the elevation at which, on average, satellite images are taken at this site^27^. This can be explained by (1) the wave runup, which in this high-energy environment can largely increase the waterline elevation^27^. Noteworthy, the two drops in correlation at $L \approx$ 20 km and 45 km correspond to the inclusion of the Cap Ferret sand spit and La Teste beaches in the south, which both show dynamics that largely contrast with that at Truc Vert^29^ due to the presence of the Arcachon tidal inlet^29,76^. We therefore consider this comparison as a fair validation of the SM SDS dataset for reasonably straight open coasts. However, agreement is expected to worsen along rugged coasts, estuary mouths, spits, islands, etc., where shoreline response is more variable alongshore, and is expected to vary depending on beach state and beach profile shape^28^.

### SDS post-processing

#### Shoreline moving averaging

Previous work shows that a spatial moving average can help reducing SDS uncertainties on open coasts^29^. However, on complex coastlines, a moving average based on distance only is not appropriate as shoreline evolution may be averaged across coastline with opposing orientation and, likely, different response modes, as would be the case for a small island for example. To avoid such a problem, here for a given transect averaging was performed across all the transects located at a distance smaller than *L*/2 and with coastline orientation $\theta$ within $\pm \delta \theta /2$ of the current transect. This allows representing rapidly alongshore variable response modes (see for instance the islands in Fig. 6d). Based on sensitivity analysis and validation at Truc Vert beach above, the computations shown in this manuscript used *L* = 5 km and $\delta \theta$ = 45$^\circ$.

#### Long-term shoreline trend

Long-term rates of shoreline change were calculated for 1984–2021 using linear regression. For this, we averaged spatially the time series of shoreline position around the mean for a group of transects. This group of transect was based on e.g. latitude bins and coastline orientation (Fig. 3b–e), coastline exposure *D* (in Fig. 2a), geographic area (see validation in Fig. 8) or the moving-averaged approached described above based using *L* and $\delta \theta$. The fair comparison of the SM SDS computed trends against in situ or state-of-the art shoreline dataset above provide an uncertainty of ±0.4 m/year for all the computed long-term trends.

#### Shoreline change correlation with climate indices

Consistent with previous work showing at more local scale that interannual shoreline variability can be related with some large-scale climate modes of atmospheric variability in winter^29,43^, we compared *dS* the yearly change in shoreline position with the dominant climate indices. We used the winter-mean (DJFM) values of the conventional teleconnection indices NAO, SCAND and EA, which show links with wave climate variability along the Atlantic coast of Europe (e.g.^62,77^), and with shoreline response locally (e.g.^29,33,43^). These indices are computed from Empirical Orthogonal Function (EOF) analysis of the sea level pressure field. In addition, we also used the WEPA index which, in contrast with NAO, SCAND and EA, was specifically designed to explain the winter-mean wave height variability along the Atlantic coast of Europe where the NAO and other indices showed poor correlation. Noteworthy, this index also skilfully explains the interannual variability in e.g., winter-mean precipitation, river discharge, coastal water temperature and salinity of coastal water in western Europe (e.g.^78,79^). Winter WEPA time series was computed by normalising (1942–2021) the in situ sea level pressure difference measured at the Valentia and Santa Cruz de Tenerife weather stations. The (yearly) time series of moving-averaged shoreline position around the mean was computed as well as its yearly change time series *dS*. Correlations and corresponding p-values between *dS* and all the climate indices were then computed to explore which climate indices explain some amount of interannual shoreline variability, and how these correlations vary spatially. Pearson correlation coefficients measuring the linear relationships were used, showing very similar results to nonlinear correlation (e.g. Spearman).

## Data Availability

The datasets generated and further analysed during the current study are available in the Open Science Framework repository, https://osf.io/jftw8/.
